# Molecular Signatures of High-Grade Cervical Lesions

**DOI:** 10.3389/fonc.2018.00099

**Published:** 2018-04-12

**Authors:** Andreia M. Porcari, Fernanda Negrão, Guilherme Lucas Tripodi, Denise Rocha Pitta, Elisabete Aparecida Campos, Douglas Munhoz Montis, Aline M. A. Martins, Marcos N. Eberlin, Sophie F. M. Derchain

**Affiliations:** ^1^Thomson Mass Spectrometry Laboratory, Department of Chemistry, University of Campinas, UNICAMP SP, Campinas, Brazil; ^2^Department of Obstetrics and Gynecology, University of Campinas, UNICAMP SP, Campinas, Brazil; ^3^Brazilian Center for Protein Research – LBPQ, Medicine College, University of Brasília, Brasília, Brazil

**Keywords:** human papilloma virus screening, metabolomics, mass spectrometry, cervical cancer, cervical cytologic specimens, translational research, pre-neoplastic phenotype, molecular signatures

## Abstract

Cervical cancer is the fourth most common neoplasia in women and the infection with human papilloma virus (HPV) is its necessary cause. Screening methods, currently based on cytology and HPV DNA tests, display low specificity/sensitivity, reducing the efficacy of cervical cancer screening programs. Herein, molecular signatures of cervical cytologic specimens revealed by liquid chromatography-mass spectrometry (LC-MS), were tested in their ability to provide a metabolomic screening for cervical cancer. These molecules were tested whether they could clinically differentiate insignificant HPV infections from precancerous lesions. For that, high-grade squamous intraepithelial lesions (HSIL)-related metabolites were compared to those of no cervical lesions in women with and without HPV infection. Samples were collected from women diagnosed with normal cervix (*N* = 40) and from those detected with HSIL from cytology and colposcopy (*N* = 40). Liquid-based cytology diagnosis, DNA HPV-detection test, and LC-MS analysis were carried out for all the samples. The same sample, in a customized collection medium, could be used for all the diagnostic techniques employed here. The metabolomic profile of cervical cancer provided by LC-MS was found to indicate unique molecular signatures for HSIL, being two ceramides and a sphingosine metabolite. These molecules occurred independently of women’s HPV status and could be related to the pre-neoplastic phenotype. Statistical models based on such findings could correctly discriminate and classify HSIL and no cervical lesion women. The results showcase the potential of LC-MS as an emerging technology for clinical use in cervical cancer screening, although further validation with a larger sample set is still necessary.

## Introduction

Cervical cancer is the fourth most common neoplasia in women, and the seventh overall. In 2012, for instance, an estimative of 528,000 new cases and 266,000 deaths from cervical cancer were reported worldwide, accounting for 7.5% of all female cancer deaths. Almost 9 out of 10 cervical cancer deaths occur in the less developed countries due to poor effectiveness of screening, lack of early detection, and treatment of precancer (1, 2). In Latin American and Caribbean, cervix cancer has been the second highest cause of mortality among women (1). In Brazil, the last survey highlighted cervical cancer as the fourth most common cancer with 7.9% incidence and the projections for the biannual period 2016–2017 is the incidence of 47,400 new cases (2).

Infection with human papilloma virus (HPV) is recognized as the cause of invasive cervical cancer (3). There are more than 200 known HPV types classified according to their carcinogenic potential in high-risk and low-risk HPV (4). The risk depends on whether the virus will develop a productive infection by achieving basal or parabasal cell layers of the epithelium or whether the virus will infect the specialized stem cell populations at the transformation zone, making a transformative infection. Productive infections are transient, become undetectable after several months, and do not lead to cancer, whereas transformative infections are persistent over many years and can lead to precancer lesions and cervical cancer. Among the high-risk HPV, HPV16 and HPV18 are responsible for 70% of all the cervical cancer cases (3, 5). Cervical lesions generated by HPV infection are classified according to lower anogenital squamous terminology (LAST) as those leading to low-grade squamous intraepithelial lesions (LSIL) or high-grade squamous intraepithelial lesions (HSIL) (6). About 75% of women infected with high-risk HPV display normal cytological features and present no lesions (4).

The primary goal of cervical cancer screening programs is, therefore, to prevent cervical cancer by detecting and treating cancer precursors (7). But, an efficient screening program needs to be sufficiently accurate and acceptable to the target population and comprises the triage of screen-positive women to determine who among them requires colposcopy (cervical examination) or treatment. Currently, there are two major alternatives for cervical cancer screening: cytology and DNA tests for the detection of high-risk HPV types (4). Cytology screening is expected to display better performance for triage of HPV-positive women compared to primary cytological screening (8). Controversial findings about the sensitivity of cytology are, however, not surprising given the subjective character of this screening test (9). Besides cytological examination and testing for high-risk HPV, there is a continuing need for viral and/or host markers of disease that will help to distinguish clinically insignificant, self-limited HPV infections from precancerous infections (4, 5).

The subjective composition of cytology results among HPV-positive women calls for alternatives that include automated screening methods. Such automation would allow the development of integrated approaches using HPV testing and automated tests, minimizing the subjectivity of cervical cancer screening (8). Among the analytical tools that are now available, mass spectrometry (MS) particularly when combined with separation strategies, such as liquid chromatography (LC), is an emerging technology that enables the detection, quantification, and characterization of multiple analytes in the same test, in an untargeted or targeted way (10, 11). The use of MS or LC-MS is actual in clinical laboratory for many diagnoses, such as to detect inborn errors of metabolism (12, 13), analysis of steroid hormones (14), microbial identifications (15, 16), cancer diagnosis, and surgical margin evaluation (17). LC-MS is also very suitable for metabolomics studies, since it generates rich biomolecular profiles derived from specific cellular metabolisms, thus providing detailed insights of the biochemistry during cellular pathophysiological changes (18). Compared to genomic and proteomics, metabolomics approaches provide the clearest molecular picture of the phenotype of a biological system, since metabolites are the end products from both gene transcription and translation (proteins) (19), therefore, reflecting and complementing most relevant biological information from primary metabolism.

In the search for diagnosis biomarkers for cervical cancer, investigations over proteomics content have been performed from cervical tissue, directly over the tumor and using cervical cytologic species (20–27). For metabolomics approaches, few studies using urine (28, 29) or plasma and serum samples (30–32) have been performed aiming at investigating intraepithelial lesions. Cervical cytologic specimens have also been recently analyzed in a preliminary study using MS, aiming at differentiating early-stage precancer lesions (i.e., LSIL) and high-risk HPV persistence, and their results reinforce the potential of investigating metabolites alterations as diagnosis biomarkers (33). The recurrent cicatrization, due to pathogen-host wound-healing response, leads indeed to important changes in microenvironment effectors, favoring basal membrane reinfection (34). These events are much closely related to metabolic reprogramming and metabolites alterations (35).

Herein, we tested the ability of LC-MS to perform an efficient metabolomics screening of cervical cancer *via* molecular signatures. We tested whether the LC-MS methodology would be able to differentiate clinically insignificant HPV infections from precancerous lesions. HSIL-related metabolites were also compared to those of no cervical lesions in women with and without HPV infection. Indeed, the LC-MS method, using the same cervical cytologic specimen employed for cytology and DNA tests, was found to efficiently screen for cytologic abnormalities and to detect for molecular signatures related to the pre-neoplastic phenotype, which helps in the understanding of the molecular mechanisms underlying cervical cancer predisposition.

## Materials and Methods

### Clinical Specimens

Cervical cytologic samples were obtained from women from two groups: (i) 40 women undergoing gynecological examination in the family planning clinic (FPC) from the Women’s Hospital “Prof. Dr. José Aristodemo Pinotti”—CAISM-UNICAMP, previously diagnosed with normal cervix in the clinical evaluation and (ii) 40 women undergoing to a loop electrosurgical excision procedure (LEEP) due to previous detected HSIL from cytology and colposcopy, in the surgical service from CAISM-UNICAMP. Women in both group had their sample collected prior to cervical manipulation and following the same collection instruction, by trained personnel. For cervix diagnosis, the following information was considered: (i) clinical evaluation at collection; (ii) liquid-based cytology diagnosis; (iii) HPV test results; and (iv) histology evaluation of the excised tissue from HSIL patients. Inclusion criteria were clinically normal cervix with negative cytology for the “no cervical lesion (NCL) group” and HSIL confirmed lesion from histology evaluation of the transformation zone for the “HSIL group.”

This study was carried out in accordance with the recommendations of the Resolution no. 466/2012 of the Brazilian National Health Council (CNS), with written informed consent from all subjects and in accordance with the Brazilian National Research Ethics Commission (CONEP). The protocol was approved by the Research Ethics Committee of the University of Campinas (CEP/UNICAMP) under the number 1.181.203 from 08/25/2015. Specimens were collected in a laboratory-made, preservative-free liquid-based preparation (10 mL of ethanol:water, 75:25,% v/v) (36). Cervical scrapings were taken in duplicate by cytobrush (Surepath^®^, BD) and immediately immersed in the same collection vial containing the ethanolic solution. After homogenization, each sample was divided as follows: (i) 50% of the material was sent to chemical extraction for LC-MS analysis; (ii) 10% was dedicated to preparing liquid-based cytology slides to be diagnosed by a board-certified pathologist; and (iii) the remaining 40% was sent to the Laboratory of Molecular Biology at CAISM to screen for HPV DNA. Maximum interval of 72 h was considered between sample collection and preparation prior to further extraction procedures.

### Sample Preparation for Liquid-Based Cytology

Cervical samples in solution were gently vortexed and a volume ranging from 0.5–1 mL was manually dripped on a silanized glass slide. The slides were allowed to dry, sent to the Cytology Laboratory at CAISM (UNICAMP), and then prepared using standard protocols for cytology. These steps were done in duplicate resulting in two slides per sample which were analyzed by a board-certified pathologist.

### Squamous Cellularity Estimation

All the slides evaluated by the pathologist had their number of squamous cells estimated according to the Bethesda System. This estimation was obtained by performing representative field cell counts. For that, four adjacent microscopic fields were counted per slide at ×40 magnification and their results were averaged. The correlation of 3.8 visualized cells for 5,000 estimated cells was used. Such estimative showed the adequacy of the specimens for cytology analysis. Correlation studies of cell counting and relevant clinical variables were also examined to investigate possible bias added to the results which could be related to the contraceptive method of choice, menstrual cycle phase, age, and body mass index of the subjects. Figures S1–S4 in Supplementary Material show box plots of cell counting correlating to these variables. None of the analyzed variables showed significant differences for this test set.

### Sample Preparation for HPV Detection

A part of the original specimen solution (about 4 mL) was centrifuged (6000 RPM, 10 min). The resulting pellet was transferred to a 1.5 mL microtube and centrifuged again (1900 RPM, 10 min). The cellular pellet was kept frozen at −20^o^C until DNA extraction. DNA extraction and quantitation were performed as previously described, as well as β-globin amplification serving as internal control for quality and sufficiency of sample’s DNA (37). HPV DNA detection was amplified in replicate tubes using the L1 consensus primers PGMY 09 and PGMY 11, primarily designed to detect α-HPVs (i.e., α-papillomavirus, predominantly isolated from mucosal and genital lesions) (38, 39). Specific HPV types were not determined in the HPV-positive PCR samples. During each polymerase chain reaction (PCR) run, all samples were tested together with one negative control (water) and one positive control (HPV 18-containing cells) (40).

### Sample Preparation for LC-MS Analysis

From the original cervical sample, 5 mL were vortexed and transferred to a new vial followed by the addition of 10 mL of water (Milli-Q). After this, vials were centrifuged (5000 RPM, 10 min) and the supernatant was discarded. Water (800 μL) was added to the pellet and the cellular content was then transferred to a 2 mL microtube, followed by the addition of methyl tert-butyl ether (MTBE, 1 mL). Samples were then submitted to an automatic vortex mixer (10 min) and centrifuged (14000 RPM, 10 min). The upper layer was collected, sent to vacuum centrifugation until dryness and stored at −20°C until analysis, when these extracts were resuspended in 120 μL of a methanol:chloroform solution (2:1,% v/v).

### LC-MS Analysis and Data Extraction

Data acquisition was performed using a LC-MS system composed by an HLPC Agilent 1290 Series equipped with an Acquity C18 column, 2.1 × 100 mm, 1.7 μm (Waters). The injection volume was 10 μL, the oven was kept at 40^o^C and a flow rate of 600 μL min^−1^ was used. The composition of the mobile phase is shown in Supporting Table S1 in Supplementary Material. HPLC was coupled to a hybrid 6550 Quadrupole-Time-Of-Flight Mass Spectrometer (Q-TOF-MS, Agilent) equipped with an electrospray (ESI) ionization source. Samples were analyzed in the positive ion mode, using the following instrumental parameters: V*Cap* 2.800 V; fragmentor voltage at 175 V; skimmer voltage at 60 V; OCT 1RF Vpp at 750 V; gas temperature at 260^°^C; sheath gas temperature at 300^°^C; and drying gas at 12 Lmin^−1^. Mass spectra were acquired in centroid mode and the acquisition mass range was 100–1,500 Da. Samples were randomly analyzed. Raw data were converted to the file format *.mzData* at MassHunter Qualitative software (Agilent), using filters of ions for relative intensity of 5% of the most intense ion. Files were then imported to the software XCMS online (https://xcmsonline.scripps.edu/) for retention time alignment and for the extraction of ion chromatograms (EIC) (41). Main parameters used to build the XCMS alignment method are shown in Supporting Table S2 in Supplementary Material. XCMS provides a table containing the ions labeled according to their nominal masses and retention times in function of the EIC intensity for each sample, which was utilized to the statistical analysis.

### Statistical Analysis and Biomarkers Selection

Statistical analysis was performed through multivariate models available in the MetaboAnalyst website (http://www.metaboanalyst.ca) (42). No data filtering was used. Data were normalized by sum and *Pareto* was used as data scaling. For the unsupervised analysis, principal component analysis (PCA) was used. For the supervised analysis through support vector machine (SVM), a total of 70% of the available data were used to build a statistical method of classification (training set), whereas about 30% of the available data was used as an independent test set. Receiver operating-characteristic (ROC) curves were calculated for the performance of SVM models and the models with the highest area under the ROC curve (AUC) value were selected as the optimal ones. Potential biomarkers were identified through supervised statistical analysis by SVM and the minimal number of ions which could discriminate the groups was selected to compose the statistical model.

### MS/MS Experiments and Biomarker Assignments

Ions indicated as those formed from putative biomarkers by the statistical analysis were selected for MS/MS experiments and for that, a Q-TOF 6550 (Agilent) instrument was used. For ion assignment, the exact mass of the ion and its fragments were considered and then compared to databases, such as Lipidmaps (http://www.lipidmaps.org) and Metlin (https://metlin.scripps.edu).

## Results

### Subjects and Their Diagnosis Through Cytology, HPV Screening, and Histology

A total of 80 subjects were recruited and gave their formal consent. Five of these subjects had their sample excluded from the study due to unsatisfactory cellularity and/or absence of endocervical/metaplastical cells (43) for cytology evaluation or presence of confirmed carcinoma through histology. Tables S3, S4 in Supplementary Material present detailed information on each sample diagnosis.

Table 1 shows a summary of diagnosis for HPV-positive and HPV negative women. Considering the results for cytology and histology for HSIL subjects, there was an agreement of 91% (35 HSIL subjects confirmed *via* histology). This agreement shows the adequacy of sample preparation for cytology, which was adapted to use a preservative-free liquid-based medium for collection of specimens. HPV was detected in 32 (91%) of women from HSIL group compared with 23 women (58%) with normal cervix. The normal cervix group was composed of a sexually active young population, attending to a family planning clinic, thus justifying their risk factor for HPV infection (5). Results for HPV detection were also satisfactory, since all the samples showed adequate results for β-globin amplification, used as internal control, showing, therefore, the adequacy of the preservative-free medium for DNA preservation. The use of this customized medium was determinant for LC-MS analysis, since other commercial media were tested and showed a high content of unwanted polymers and other interferents (data not shown) (33).

**Table 1:** Summary of subjects, their attributes, and results for cytology, human papilloma virus (HPV) screening, and histology procedures.

Parameter	HPV positive (*N* = 55 subjects)	HPV negative (*N* = 20 subjects)
Age	34 ±8 years	36 ± 13 years
Age at first sexual intercourse	16 ± 2 years	16 ± 2 years
Sexually active interval	19 ± 8	19 ± 11
Body mass index	28 ± 5^a^	28 ± 7
Cytology for NILM	24	19
Cytology for high-grade squamous intraepithelial lesions (HSIL)	31	1
Histology for HSIL	32	3

*^a^Calculated over 47 subjects; 8 missing data*.

*NILM, negative for intraepithelial lesion or malignancy*.

### LC-MS Results

For the differentiation of HPV-positive subjects, two groups were considered for SVM analysis: (i) normal cervix subjects screened positively for HPV, designed as the *NCL*+ group and (ii) HSIL subjects screened positively for HPV, designed as the *HSIL*+ group. The aim of this comparison was to find whether the metabolomic profiles of these samples would reveal differences assigned to the precancer stage (HSIL). For that, SVM was applied for training and test sets.

Support vector machine is a multivariate classification algorithm using a non-parametric machine learning technique, which identifies important variables for the construction of both classification and regression models. It was developed to solve binary problems, such as case–control studies and, compared to other multivariate classification methods, SVM is less susceptible to outliers and over-fitting (11, 19).

**Figure 1:**
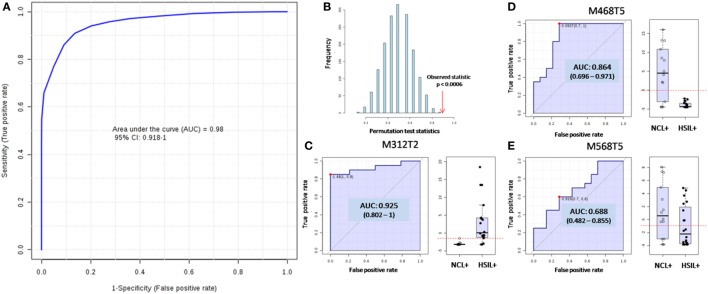
Supporting vector machine (SVM) model for differentiation of women screened positive for human papilloma virus (HPV) and who were diagnosed (i) with high-grade squamous intraepithelial lesions (*HSIL*+ group) and (ii) with no cervical lesion (*NCL*+ group) according to metabolomics. **(A)** The receiving operating-characteristic (ROC) curve plotting the true positive rate against the false positive rate for the model as a whole, which displayed an area under the ROC curve (AUC) of 0.98 for the test set, with average accuracy of 89.4% based on 100 cross validations. **(B)** The *p*-value was found to be significant since it is less than 0.05. The individuals ROC for the three molecules used for building the model and the relative abundances distribution of these molecules over the groups shown by the box-plots are displayed for M312T2 **(C)**, M468T5 **(D)**, and M568T5 **(E)**.

Figure 1A shows the ROC curve, which plots the sensitivity (true positive rate) as function of 1-specificity (false positive rate, for a 95% confidence interval—CI). The area under the ROC curve (AUC), which is a measure of how well a parameter can distinguish between two diagnostic groups was found to be 0.98 in the optimal model. The average accuracy based on 100 cross validations was found to be 89.4%. Figure 1B shows the *p*-value for such differentiation, which is significant when it is less than 0.05. The resulting SVM model was applied to classify the test set and was fortunately found to correctly classify 8 out of 9 *HSIL*+ tested samples and 6 out of 6 *NCL*+ samples which had not been used before to build the statistical model, resulting in a positive predictive value (PPV) of 1.00, negative predictive value (NPV) of 0.86, specificity of 1.00, and sensitivity of 0.89 in a per-patient analysis. The medium probability of correct classification found for the test set was 81.3% for *HSIL*+ group and 86.4% for *NCL*+ group.

The model was built based on three molecular features: (i) the ion of *m/z* 312.326 eluted with mean retention time (mRT) of 1.64 min and termed M312T2; (ii) the ion of *m/z* 468.440 eluted with mRT of 4.89 min and termed M468T5; and (iii) the ion of *m/z* 568.445 eluted with mRT of 5.22 min and termed M568T5. Figures 1C–E show the individual ROC of these features, as well as their distribution over the groups in terms of relative abundances. These ions were tentatively assigned based on their exact *m/z* values and their fragmentation behavior (Figures S5, S6 in Supplementary Material). The feature M312T2 was assigned as the protonated molecule [M − H_2_O + H]^+^ of dimethyl sphinganine (d18:0), a sphingosine metabolite. The feature M468T5 was assigned as the protonated molecule [M − H_2_O + H]^+^ of the Ceramide CE (29:1). The feature M568T5 could not be firmly characterized, but it was assigned as the protonated molecule [M + H]^+^ of a derivative of a ceramide, since its fragmentation profile is similar to the one observed to M468T5.

A comparison among HPV-negative subjects, containing or not HSIL lesions, was also performed. Figure S7 in Supplementary Material shows the results and, although in a reduced number of samples for HSIL/HPV-negative subjects (*N* = 3, *HSIL−* group), the same three molecular features mentioned above were also able to correctly discriminate the groups with an excellent average accuracy of 100%. This comparison confirmed the previous obtained results for HPV-positive subjects, since the same set of molecular features was used to discriminate the groups containing or not the precancer lesion.

**Figure 2:**
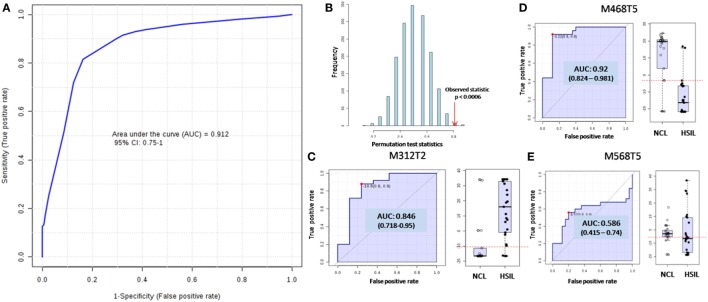
Supporting vector machine (SVM) model for differentiation of high-grade squamous intraepithelial lesions (*HSIL*) group and no cervical lesion (*NCL*) group according to their metabolomics and clinical evaluation at collection time. **(A)** The receiving operating-characteristic (ROC) curve ploting the true positive rate against the false positive rate for the model as a whole, which displayed an area under the ROC curve (AUC) of 0.91 for the test set, with average accuracy of 80.9%, based on 100 cross validations. **(B)** The *p*-value was found to be significant since it is less than 0.05. The individuals ROC for the three molecules used for building the model and the relative abundances distribution of these molecules over the groups shown by the box-plots are displayed for M312T2 **(C)**, M468T5 **(D)**, and M568T5 **(E)**.

A last comparison model was built with LC-MS data based only in the clinical evaluation of the subjects at the moment of sample collection, without reference to their HPV status, cytology results, or cell counting/sample quality. The *HSIL* group was composed of 35 subjects, whereas the *NCL* group was composed of 40 subjects. Applying the SVM method in a training set of 50 samples, the AUC was found to be 0.91, with *p* < 0.05 (Figures 2A,B). The average overall accuracy based on 100 cross validations was found to be 80.9%. For the 28 samples used as test samples, only 3 *NCL* were misclassified, resulting in a PPV of 0.81, NPV of 1.00, specificity of 0.80, and sensitivity of 1.00 in a per-patient analysis. The medium probability of correct classification found for the test set was 78.8% for *HSIL* group and 88.7% for *NCL* group. The model was built based on the same three molecular features previously found and Figures 2C–E show their contribution to the model.

Considering the site of sample collection (FPC or LEEP operating room), unsupervised analysis of the data was also performed through PCA (Figure S8 in Supplementary Material). The analysis shows reproducibility among samples considering the site of collection as a variable. The procedures carried out at different sites, therefore, were equivalent and failed to interfere with the results.

## Discussion

In this study, we found that there is a proper collection medium for liquid-based cytology that enables to analyze (i) cytology, (ii) HPV DNA screening, and (iii) metabolomics through LC-MS, all performed over the same sample, without the need of extra sample collection and without loss of quality for cytology and HPV screening. The correlation of the results of these three techniques, summed to the clinical evaluation of the subjects and to the histology results of excised specimens from *HSIL* group, allowed us to build an efficient model using the metabolomic data of the samples. This model was found to discriminate and correctly classify 93% of HPV-positive subjects presenting HSIL from those with no cervical lesion for a test set of 15 samples. Three molecular features, assigned to sphingolipids, were found to be the most discriminant and, therefore, were selected to build the model. We used this set of biomolecules also to discriminate HPV-negative subjects presenting HSIL from those with no cervical lesion. When performing a blind test using only LC-MS to differentiate histologically confirmed *HSIL* and *NCL* women, a high discrimination power was found: that is 25 out of 28 samples were correctly classified. Although our analysis was performed using LC-MS, which could provide quantitative assessment of the molecular markers, in our approach we used statistical analysis to obtain a diagnosis based on the detected levels of the predictive features, which provided high accuracy for cancer detection. These results showcase the potential of this technology for clinical use in cervical cancer screening, since it can correctly detect and diagnose HSIL in patients, independently of their HPV status or cytological evaluation, although further validation with a larger sample set remains necessary.

This study emphasizes the importance of interdisciplinary investigations and translational studies in finding prognostic markers that could solve the insufficient specificity and sensitivity of present cervical cancer screening programs from the molecular point of view (7, 44, 45). Studying the molecular signatures of cervical lesions would lead to more selective and sensitive techniques helping to better understand the progression of such lethal and so widely spread disease.

In this study, the concurrent use of these two main screening techniques with clinical examination (for all the subjects) and histological evaluation of excised specimens (for HSIL subjects) ensured the proper diagnosis of samples (8). The LC-MS metabolite data could then be used to create and calibrate a reliable model (46, 47). The proposed LC-MS method has shown to be compatible with both HPV screening and cytology, since the collection medium of choice offered the possibility of analyzing the samples using these three techniques, without the loss of quality in analysis. After further validation studies, LC-MS could also be automated to achieve a high-throughput status for sample management, as widely exemplified in other tests using this methodology for clinical diagnosis (48, 49).

The LC-MS method coupled to SVM statistical analysis satisfactorily discriminated HPV-positive subjects with or without HSIL. Although it is not a progressive study, since such a tremendous ability would require further studies and the follow-up of a group of patients for several years/decades, the results found here do present a plausible perspective, since unique molecular signatures able to discriminate precancer lesions were found in the phenotype of HSIL patients rather than in their genotype, since both *HSIL*+ and *NCL*+ group were composed of HPV-positive subjects. The model has been already demonstrated to be able to classify unknown samples assigning the correct diagnosis with good statistical results. The minimum number of characterized molecular features was selected to build the model, so as to ensure that these features had some biological relevance and that they were not contaminants or artifacts of the technique. To study the mechanism of disease progression, most if not all the detected features should be considered and characterized, thus generating a panel of molecules which could enable the illustration of the biological processes taking part in HSIL formation and/or HPV infection, which was not the aim of this study (50).

The three molecular features found as relevant for the model are related to disruptions in sphingolipids metabolism, hence they may be involved with pathogen–host cellular transformations (51–53) or inducing apoptosis and cellular autophagy (54). Due to pathogen–host cellular transformation, sphingolipids play an important role on lipid rafts in cell membrane (51, 52), which are associated with cellular microbial response (53, 55). Ceramides are also key players on this event, as ceramide can be generated by *de novo* synthesis or hydrolysis of sphingomyelin by sphingomyelinases (56, 57) on lipid rafts. In fact, ceramides participate on relevant cellular events as differentiation processes (58), pathogen–host immune response (59), apoptosis and cell cycle arrest (60), and senescence (53). The molecular signatures found in this study are, therefore, in accordance with observed events linked to sphingolipids metabolism, moreover to ceramides regime. The HSIL formation seems to affect ceramide synthase pathway, altering the cell regulation and increasing cellular sphingoid bases concentration (like dimethyl sphinganine). This pathway affects also complex sphingolipids and may decrease ceramide biosynthesis, as exemplified through the lower abundance of CE(29:1) and M568T5 found in HSIL samples. Cells sensitive to the proliferative effect of decreased ceramides and increased sphingoid bases may be selected to survive and proliferate and, when the cells selected to survive are abnormal, then cancer risk may increase (61, 62). A decreased ceramide level has already been observed in malignant cells resistant to apoptosis (63) and these findings are in accordance with the previous observed for metastatic colorectal cancer (64–66). Other analytes could be found if our study was performed in dedicated samples (32, 67). However, one of the aims of our study was the alignment of MS protocols to the already existing clinical routines, allowing the investigation of new biomarkers on the “waste” of other very well-established techniques, such as cytology and HPV test. For that, cervical cytological species were chosen to create a three-in-one methodology, including cytology, HPV test, and metabolomics profiling—over the same sample, using the same clinical proceeding, without routine alterations. For this reason, sample’s cellularity was much below optimal thresholds for MS analysis, thus resulting in low detection signals. Even though, other analytes were indeed found, but they were not pointed out as discriminant of the studied groups and, for this reason, they were not mentioned here.

The comparison among HPV-negative subjects containing or not HSIL confirmed the previous obtained results for HPV-positive subjects, since the same set of molecular features was used to discriminate the groups containing or not the precancer lesion. Again, it confirms that these changes are related to subject’s phenotype more than to their genotype. The occurrence of HPV-negative patients who had HSIL shows that the detection limit of HPV in the employed HPV test could also possibly be insufficient for infections with a lower milieu of infected cells or even that a different type of HPV, missed by the screening test, was found (less probable). HPV clearance could also be a possible reason for HPV-negative patients who had HSIL. Viral clearance occurs prior to viral integration into the host genome (carcinogenic stage) in 80% of HPV-infected women (68), therefore, Th1 pro-inflammatory cellular response has been linked to immune clearance of HPV in the female genital tract (69). The continuous injury caused by HPV infection in a chronic inflammation *momentum* could cause increase in cellular immunity, playing a decisive role in host immune clearance (70).

The last comparison is a proof of concept, which was performed without considering information regarding HPV status or cytology results of the samples, therefore, simulating the status of unknown samples. The use of LC-MS as a stand-alone technique is not suggested but certainly, after proper validation, it could be implemented in large cohort as part of cancer prevention strategies, since we showed here that the use of the very same sample used for other screening techniques is achievable, without the need of extra collection. After further studies with a larger sample set, LC-MS could, therefore, be used as a co-test for cervical cancer screening and, when LC-MS indicates any alteration, confirmation could be done for HSIL by colposcopy or biopsy. The full development of LC-MS as a method for screening routines could, therefore, bring the specificity and sensitivity that are still missing for HPV and cervical cytology screening.

There is clearly a need for improved specificity in HPV screening programs that could discriminate women who, after HPV infection, will progress to precancer status or will clear without clinical intervention (33). There is also a lot to be learned about the biological pathways which are crucial points for determining if such an infection will succeed in invasion. Metabolomic screenings *via* LC-MS, as shown here, could provide new classification models that, when aligned with other screening techniques, would enhance the knowledge of molecular signatures that could better elucidate and discriminate disease phenotype rather than its genotype. After proper method validation and optimization focused on known predictive markers, LC-MS could also achieve the qualities of being fast, automated, and less-expensive than it is on initial research stages. By performing a target analysis, this method would be compatible to sample’s preservatives generally employed in HPV and cervical cytology collection medium, thus allowing it to be used concomitantly, without major logistic changes in routine exams and biological sampling.

## Ethics Statement

This study was carried out in accordance with the recommendations of the Resolution no. 466/2012 of the Brazilian National Health Council (CNS), with written informed consent from all subjects and in accordance with the Brazilian National Research Ethics Commission (CONEP). The protocol was approved by the Research Ethics Committee of the University of Campinas (CEP/UNICAMP) under the number 1.181.203 from 08/25/2015.

## Author Contributions

AP: methodology, data analysis and interpretation of the data, and drafting of the manuscript. FN: methodology and drafting of the manuscript. GT: MS/MS data interpretation. DP: DNA-HPV determination. EC: DNA-HPV determination. DM: cytology analysis (pathologist). AM: drafting and revision of the manuscript. ME: supervision on mass spectrometry, study design; SD: study design, clinical supervision, sample collection, analysis of the data, and revision of the manuscript.

## Conflict of Interest Statement

All authors have read the journal’s policy on disclosure of potential conflicts of interest. All authors have disclosed any financial or personal relationship with organizations that could potentially be perceived as influencing the described research.

## Supplementary Material

The supporting information includes: experimental details (mobile phase composition and gradient for LC-MS analysis, alignment parameters of the software XCMS, detailed diagnosis of all the subjects), MS/MS data, supplemental statistical model for HPV-negative women and PCA analysis for site of collection equivalence investigation.

## Abbreviations

HPV, human papilloma virus; LAST, lower anogenital squamous terminology; LSIL, low-grade squamous intraepithelial lesions; HSIL, high-grade squamous intraepithelial lesions; FPC, family planning clinic; LC-MS, liquid chromatography-mass spectrometry; LEEP, loop electrosurgical excision procedure; NCL, no cervical lesion women; NILM, negative for intraepithelial lesion or malignancy; SVM, support vector machine; PCA, principal component analysis; ROC, receiver operating-characteristic (curve); AUC, area under the ROC curve; *m/z*, mass to charge ratio of an ion.
