# Supplementary material for: Molecular Signatures of High-Grade Cervical Lesions
Source: Front Oncol. 2018 Apr 12;8:99. doi: 10.3389/fonc.2018.00099 (PMC5907284; doi:10.3389/fonc.2018.00099)
Supplement: Supplementary file 1 — The supporting information includes: experimental details (mobile phase composition and gradient for LC-MS analysis, alignment parameters of the software XCMS, detailed diagnosis of all the subjects), MS/MS data, supplemental statistical model for HPV-negative women and PCA analysis for site of collection equivalence investigation. [file Data_Sheet_1.docx]

**Supporting Information**

**METABOLOMICS APPLIED TO CERVICAL CANCER SCREENING: A NEW APPROACH USING MASS SPECTROMETRY**

Andreia M. Porcari^1*^, Fernanda Negrão^1^, Guilherme Lucas Tripodi^1^, Denise Rocha Pitta^2^, Elisabete Aparecida Campos^2^, Douglas Munhoz Montis^2^, Aline M. A. Martins^3^, Marcos N. Eberlin^1^, Sophie F. M. Derchain^2^.

^1^Thomson Mass Spectrometry Laboratory, Department of Chemistry, State University of Campinas, SP, Brazil, 13083-970

^2^Department of Obstetrics and Gynecology, State University of Campinas, SP, Brazil, 13083-881

^3^Brazilian Center for Protein Research - LBPQ, Medicine College, University of Brasília, DF, Brazil, 70910-900

** to whom correspondence should be addressed:* [*andreiaporcari@gmail.com*](mailto:andreiaporcari@gmail.com)*;*

**Table of Contents**

**Supporting Table 1.** Mobile phase composition and gradient for LC-MS analysis

**Supporting Table 2.** Alignment parameters of the software XCMS

**Supporting Table 3.** Diagnosis for subjects composing HSIL group

**Supporting Table 4.** Diagnosis for subjects composing NCL group

**Supporting Figure 1.** Box-plots showing the estimated cell counting per slide in relation to the contraceptive method of choice of the subjects.

**Supporting Figure 2.** Box-plots showing the estimated cell counting per slide in relation to the menstrual cycle phase of the subjects at the time of collection.

**Supporting Figure 3.** Box-plots showing the estimated cell counting per slide in relation to the age of the subjects.

**Supporting Figure 4.** Box-plots showing the estimated cell counting per slide in relation to the body mass index (BMC) of the subjects.

**Supporting Figure 5.** (A) MS/MS spectrum of the ion of *m/z* 312.326 named M312T2. (B) Elucidation of the main fragments of the ion.

**Supporting Figure 6.** (A) MS/MS spectrum of the ion of *m/z* 468.440 named M468T5. (B) MS/MS spectrum of the ion of *m/z* 568.445, named M568T5, showing that its fragmentation takes to common fragments observed also for M468T5. (C) Elucidation of the main fragments.

**Supporting Figure 7.** The SVM model for differentiation of *HSIL-* and *NCL-* groups according to their metabolomic data.

**Supporting Figure 8.** PCA analysis of LC-MS data considering the site of sample collection as a variable.

**Supporting Table 1.** Mobile phase composition and gradient for LC-MS analysis

| **Time (min)** | **% Water + 0,1%**  **Formic Acid** | **% ACN** |
| --- | --- | --- |
| 0 | 30 | 70 |
| 1.0 | 10 | 90 |
| 10.0 | 0 | 100 |
| 16.0 | 0 | 100 |
| 18.0 | 30 | 70 |
| 25.0 | 30 | 70 |

**Supporting Table 2.** Alignment parameters of the software XCMS

| Feature Detection | maximal m/z deviation (ppm) | 5 |
| --- | --- | --- |
|  | Minimum peak width (s) | 10 |
|  | maximum peak width (s) | 60 |
|  | Signal/Noise (S/N) threshold | 10 |
|  | mzdiff | 0.01 |
|  | prefilter intensity | 100 |
|  | Noise Filter | 10 |
|  |  |  |
| Retention Time Correction | Method | Matchedfilter |
|  | profStep | 0.1 |
| Alignment | mzwid | 0.025 |
|  | minfrac | 0.5 |
|  | Allowable retention time deviations (s) | 5 |
| Visualization | EIC width | 200 |

**Supporting Table 3.** Diagnosis for subjects composing HSIL group

| **Sample ID** | **Cytology** | **HPV DNA** | **Histology** |
| --- | --- | --- | --- |
| A1* | NA | positive | HSIL |
| A2 | LSIL | negative | HSIL |
| A3 | HSIL | positive | HSIL |
| A4 | HSIL | positive | HSIL |
| A5 | HSIL | positive | HSIL |
| A6* | NA | NA | HSIL |
| A7 | HSIL | positive | HSIL |
| A8 | HSIL | positive | HSIL |
| A9 | HSIL | positive | HSIL |
| A10 | HSIL | positive | HSIL |
| A11* | NILM | positive | negative |
| A12 | HSIL | positive | HSIL |
| A13 | HSIL | positive | HSIL |
| A14 | HSIL | positive | HSIL |
| A15 | HSIL | positive | HSIL |
| A16 | HSIL | positive | HSIL |
| A17 | HSIL | positive | HSIL |
| A18 | NILM | positive | HSIL |
| A19 | HSIL | positive | HSIL |
| A20* | NILM | positive | HSIL |
| A21 | HSIL | positive | HSIL |
| A22 | HSIL | positive | HSIL |
| A23 | HSIL | positive | HSIL |
| A24 | HSIL | positive | HSIL |
| A25 | HSIL | positive | HSIL |
| A26 | HSIL | positive | HSIL |
| A27 | HSIL | positive | HSIL |
| A28 | HSIL | positive | HSIL |
| A29 | HSIL | positive | HSIL |
| A30 | HSIL | positive | HSIL |
| A31 | HSIL | positive | HSIL |
| A32 | HSIL | negative | HSIL |
| A33* | HSIL | positive | Carcinome |
| A34 | HSIL | positive | HSIL |
| A35 | HSIL | positive | HSIL |
| A36 | HSIL | positive | HSIL |
| A37 | HSIL | positive | HSIL |
| A38 | NILM | negative | HSIL |
| A39 | HSIL | positive | HSIL |
| A40 | HSIL | positive | HSIL |

* samples excluded from the study due to experimental procedure error or sample out of inclusion criteria; NA: not available; NILM: Negative for intraepithelial lesion or malignancy; LSIL: low-grade squamous intraepithelial lesions; HSIL: high-grade squamous intraepithelial lesions.

**Supporting Table 4.** Diagnosis for subjects composing NCL group

| **Sample ID** | **Cytology** | **HPV DNA** |
| --- | --- | --- |
| N1 | NILM | negative |
| N2 | NILM | negative |
| N3 | NILM | positive |
| N4 | NILM | negative |
| N5 | NILM | positive |
| N6 | NILM | positive |
| N7 | NILM | positive |
| N8 | NILM | positive |
| N9 | NILM | negative |
| N10 | NILM | negative |
| N11 | NILM | positive |
| N12 | NILM | positive |
| N13 | NILM | positive |
| N14 | NILM | negative |
| N15 | NILM | negative |
| N16 | NILM | positive |
| N17 | NILM | positive |
| N18 | NILM | positive |
| N19 | NILM | positive |
| N20 | NILM | positive |
| N21 | NILM | positive |
| N22 | NILM | negative |
| N23 | NILM | negative |
| N24 | NILM | positive |
| N25 | NILM | negative |
| N26 | NILM | negative |
| N27 | NILM | positive |
| N28 | NILM | negative |
| N29 | NILM | negative |
| N30 | NILM | negative |
| N31 | NILM | negative |
| N32 | NILM | negative |
| N33 | NILM | positive |
| N34 | NILM | positive |
| N35 | NILM | positive |
| N36 | NILM | positive |
| N37 | NILM | positive |
| N38 | NILM | negative |
| N39 | NILM | positive |
| N40 | NILM | positive |

NILM: Negative for intraepithelial lesion or malignancy

**Supporting Figures**


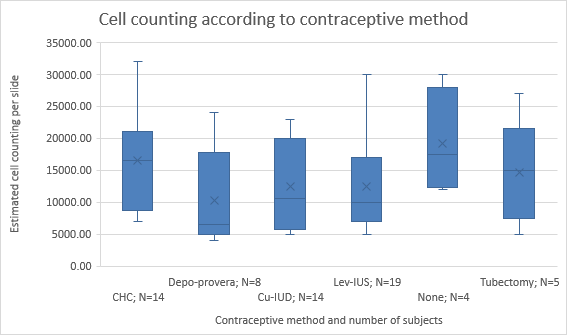


**Supporting Figure 1.** Box-plots showing the estimated cell counting per slide in relation to the contraceptive method of choice of the subjects. The methods were CHC (combined hormonal contraceptive), Depo-provera, Cu-IUD (Cooper intrauterine device), Lev-IUS (Levonorgestrel intrauterine system), none and tubectomy. Although some fluctuation on the cell counting was observed according to the contraceptive method of choice, these differences are not statistically significant for this sample set.


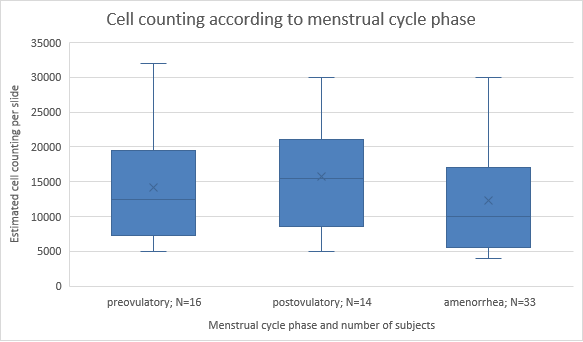


**Supporting Figure 2.** Box-plots showing the estimated cell counting per slide in relation to the menstrual cycle phase of the subjects at the time of collection. Preovulatory phase was considered until 14 days after menstruation. Postovulatory phase was considered from 15 to 42 days after menstruation. Amenorrhea was considered after 42 days from menstruation. The overall distribution shows that different menstrual cycle phases did not affect the number of cells collected from cervical smears for this study.


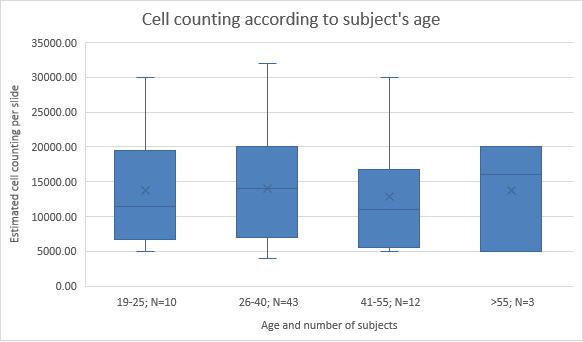


**Supporting Figure 3.** Box-plots showing the estimated cell counting per slide in relation to the age of the subjects. Subjects were divided in groups according to their age: from 19 to 25 years old, from 26 to 40 years old, from 41 to 55 years old and older than 55. The age range for this study did not significantly affect the number of cells collected from cervical smears.


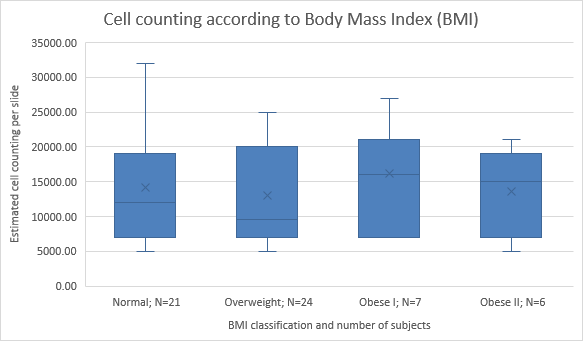


**Supporting Figure 4.** Box-plots showing the estimated cell counting per slide in relation to the body mass index (BMC) of the subjects. About 64% of the subjects have non-normal BMI. However, cell counting showed to be homogeneous in all the groups.


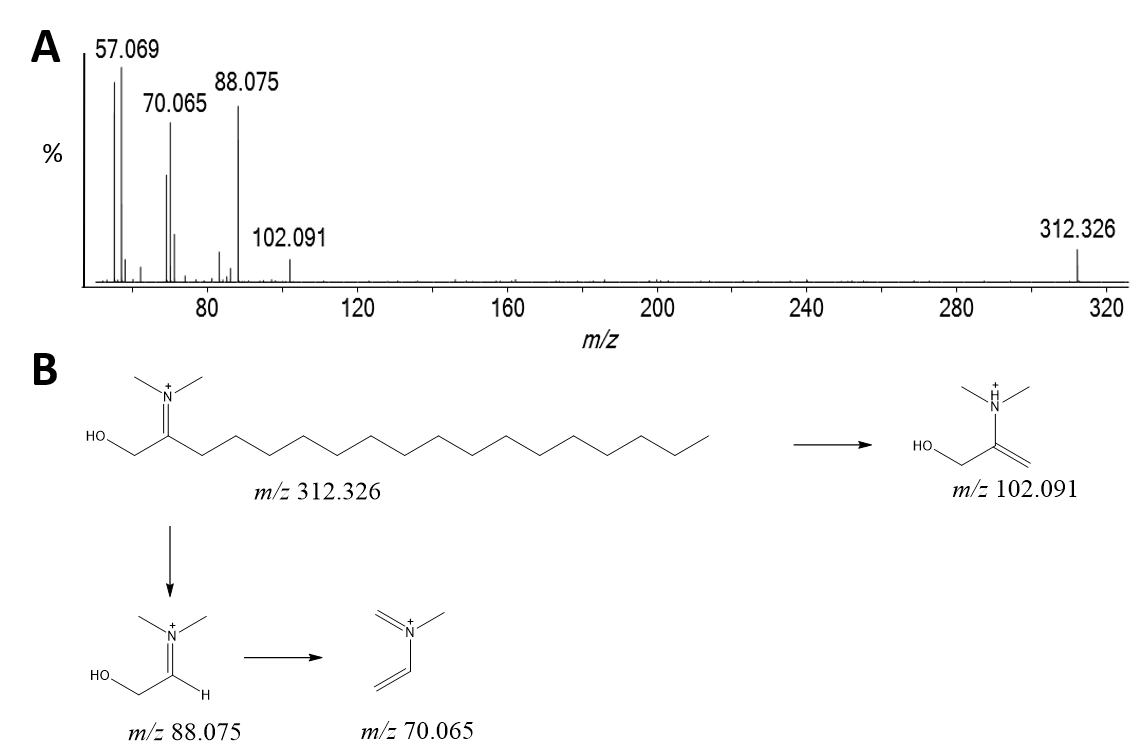


**Supporting Figure 5.** (A) MS/MS spectrum of the ion of *m/z* 312.326 named M312T2. (B) Elucidation of the main fragments of the ion.


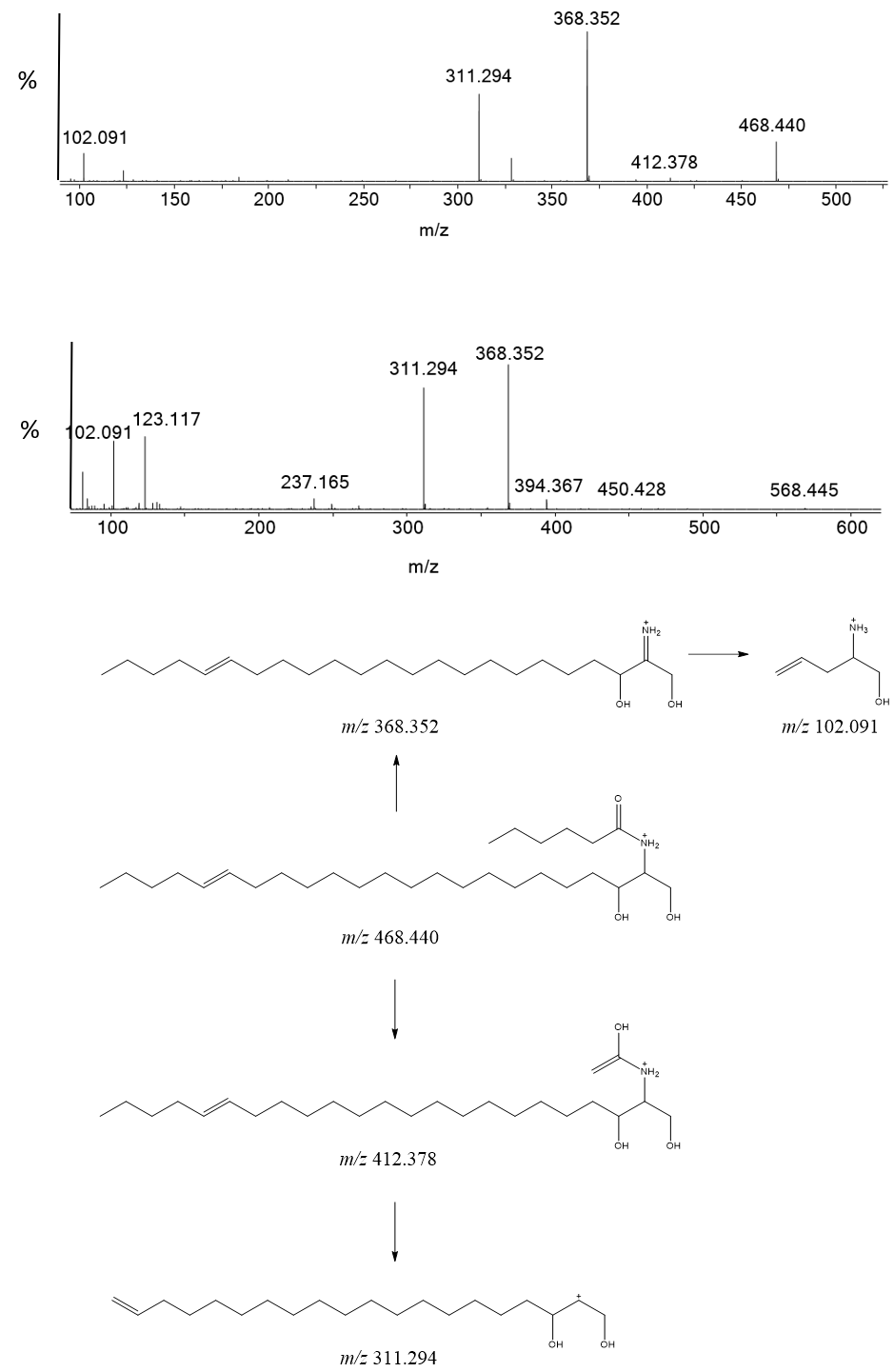


**Supporting Figure 6.** (A) MS/MS spectrum of the ion of *m/z* 468.440 named M468T5. (B) MS/MS spectrum of the ion of *m/z* 568.445, named M568T5, showing that its fragmentation takes to common fragments observed also for M468T5. (C) Elucidation of the main fragments.


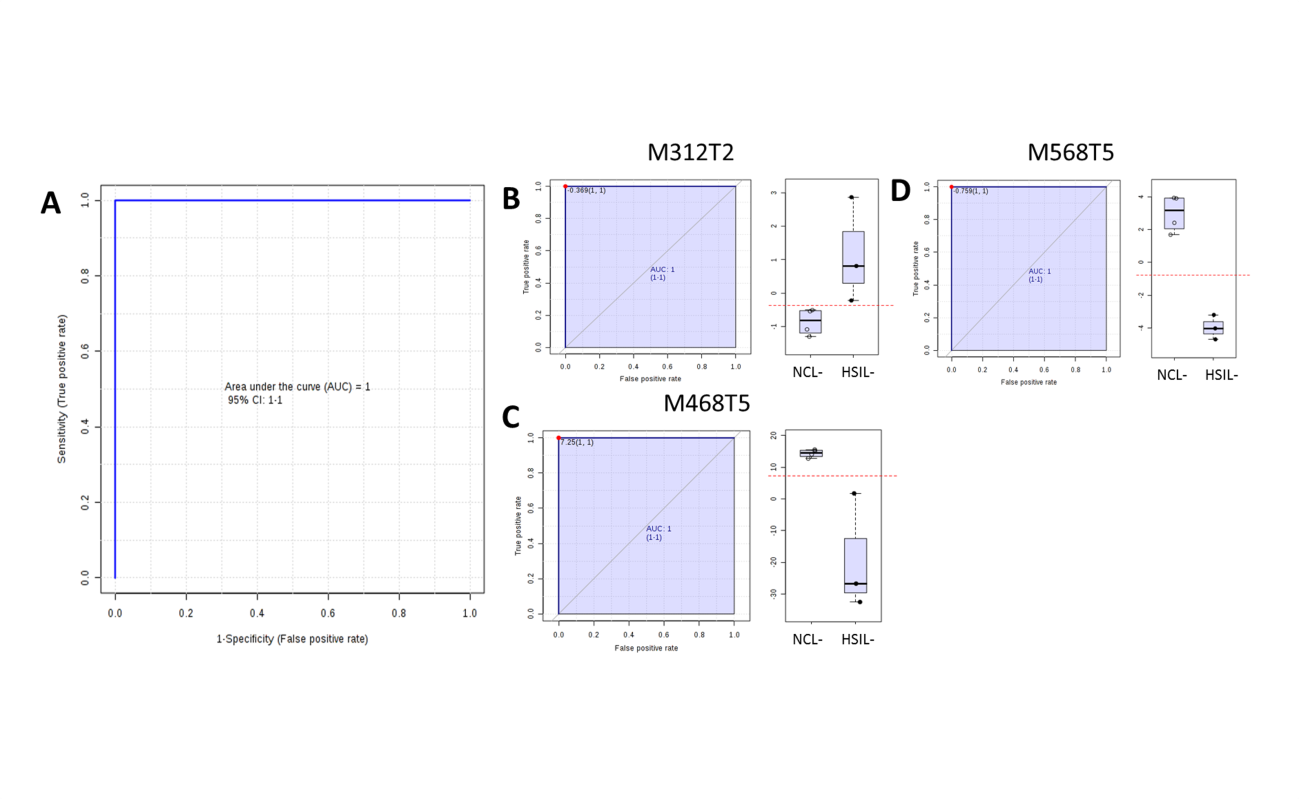


**Supporting Figure 7.** The SVM model for differentiation of *HSIL-* and *NCL-* groups according to their metabolomic data. (A) The ROC plot with AUC of 1, with average accuracy of 100%. The AUC for the three molecules used for building the model (M312T2, M468T5 and M568T5) and their relative distribution over NCL- and HSIL- groups are shown (B-D). For the comparison among HPV-negative subjects, containing or not HSIL lesions, a very limited number of subjects was available for HSIL/HPV-negative subjects (N=3, *HSIL-* group). Thus, an equivalent number of subjects HPV-negative with no lesion (normal cervix) was randomly selected to compose the *NCL-* group (N=4), in order to compose balanced groups for statistical comparison. The SVM method was also employed. No prediction or validation set was used due to the low number of *HSIL-* samples. This model was built based on the same molecular features found to be relevant for *HPV+* vs *NCL+* comparison, namely M312T5, M468T5 and M568T5.


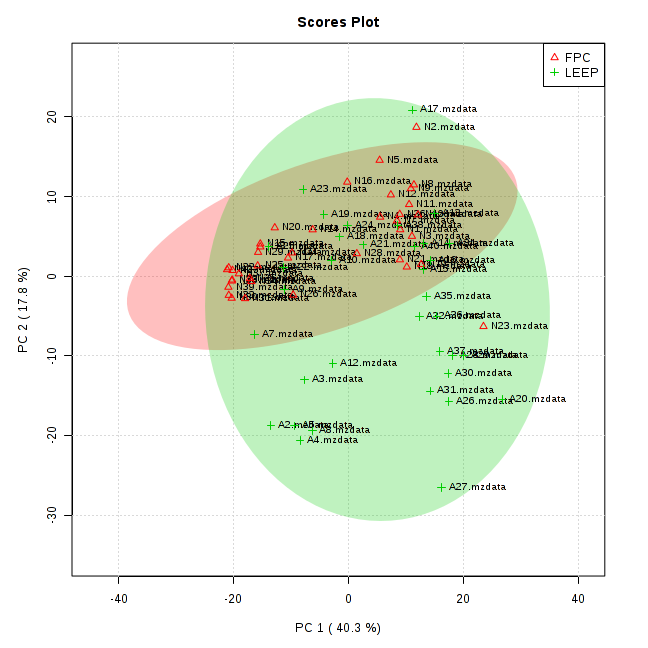


**Supporting Figure 8.** PCA analysis of LC-MS data considering the site of sample collection as a variable. FPC indicates samples collected in the Family Planning Clinic, while LEEP indicates samples collected at the loop electrosurgical excision procedure operating room. The groups could not be differentiated under unsupervised analysis, thus indicating that the collection procedures were equivalent, although performed by different people, following the same instructions.
